# Circadian feeding promotion by Ninjin’yoeito counteracts frailty in aged mice

**DOI:** 10.1016/j.jphyss.2026.100062

**Published:** 2026-01-27

**Authors:** Lei Wang, Yermek Rakhat, Masanori Nakata, Katsuya Dezaki, Hitoshi Kuwata, Boyang Zhang, Wanxin Han, Seiya Banno, Chikara Abe, Noboru Ogiso, Takashi Sakurai, Shin Tsunekawa, Daisuke Yabe, Yusaku Iwasaki, Yutaka Seino, Toshihiko Yada

**Affiliations:** a Division of Integrative Physiology, Kansai Electric Power Medical Research Institute, Osaka 553-0003, Japan; b Department of Diabetes, Endocrinology and Metabolism, Gifu University Graduate School of Medicine, Gifu 501-1194, Japan; c Kobe Biotechnology Research and Human Resource Development Center, Kobe University Graduate School of Medicine, Kobe 650-0047, Japan; d Division of Health Sciences, Medicine and Aging, Bin Zhou Polytechnic, Binzhou 256-603, China; e Department of Physiology, Faculty of Medicine, Wakayama Medical University School of Medicine, Wakayama 641-8509, Japan; f Faculty of Pharmacy, Iryo Sosei University, Iwaki, Japan; g Yutaka Seino Distinguished Center for Diabetes Research, Kansai Electric Power Medical Research Institute, Osaka 553-0003, Japan; h Department of Physiology, University of Fukui School of Medical Sciences, Fukui 910-1193, Japan; i Faculty of Health and Medical Sciences, Aichi Syukutoku University, Nagakute, 480-1197, Japan; j Department of Prevention and Care Science, Research Institute, National Center for Geriatrics and Gerontology, Aichi 474-8511, Japan; k Department of Diabetes, Endocrinology and Nutrition, Kyoto University Graduate School of Medicine, Kyoto 606-8507, Japan; l Laboratory of Animal Functional Science, Graduate School of Life and Environmental Sciences, Kyoto Prefectural University, Kyoto 606-8522, Japan; m Center for One Medicine Innovative Translational Research, Gifu University Institute for Advanced Study, Gifu 501-1194, Japan; n Department of Endocrinology, Diabetes and Metabolism, Fujita Health University School of Medicine, Aichi 470-1192, Japan

**Keywords:** Herbal medicine, Chenpi, Aging, Appetite, Pre-active phase, Anxiety

## Abstract

Frailty in aging is a major health challenge, requiring solution. Older people with frailty often exhibit malnutrition and dysregulated feeding. Feeding behavior displays circadian rhythm, while aging and frailty involve rhythm disorders, suggesting possible role of circadian feeding in frailty and treatment. Herbal medicine, Ninjin’yoeito (NYT), reportedly ameliorates frail symptoms. The present study explored impacts of NYT on circadian feeding and psychological/physical functions in aged mice. Here, we report that oral NYT independent of administration timing increases food intake specifically in 18:00–20:00, the pre-active phase, in aged and young mice, an effect mimicked by Chenpi and hesperidin. NYT altered appetite-regulating hormones and neuropeptides in pre-active phase. Repeated NYT administration restored anti-anxiety behavior, memory, and grip strength that declined in aged mice. These effects were blocked by food deprivation and pair-fed to control selectively in 18:00–20:00. These results reveal pre-active phase feeding promotion as a novel avenue to intervene aging-related frailty.

## Highlights

• Circadian feeding promotion by Ninjin’yoeito (NYT) counteracts frailty in old mice.
• Critical circadian time for feeding to counteract frailty is the pre-active phase.
• NYT alters neurohormones regulating appetite and mind in pre-active phase.
• Boosting early morning appetite and breakfast can intervene frailty in old people.
• It can be achieved with NYT as medicine and Chenpi/hesperidin as food/supplement.

## Graphical Abstract

**Figure:**
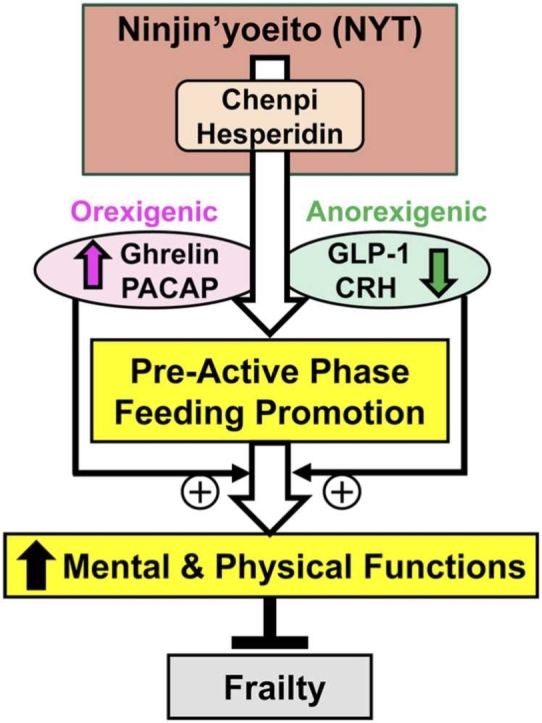

## Introduction

Frailty, the concept of integrative vulnerability in physical, psychological and/or social abilities in older people [1], [2], has become a serious problem in modern aging societies [3]. Strategies against frailty and aging are in high demand but currently unestablished. Older adults with frailty and/or sarcopenia are often undernourished and accompanied with dysregulated feeding including reduced appetite, recognized as anorexia of aging, positioning the feeding as an effective target for intervention [4], [5].

Feeding is characterized not only by quantity and quality but by timing of food intake. Physiological feeding behavior is under circadian control, recognized as circadian feeding rhythm [6], [7], and feeding regimens influence circadian rhythms [8], showing tight bidirectional link between circadian rhythm and feeding. Notably, aging and frailty involve rhythm disorders [9]. These observations suggest the circadian feeding behavior as a possible cause and/or therapeutic target for frailty. However, it has not been verified, and moreover the specific circadian time when food intake has particular impact on frailty remains unknown. It has been reported that the treatment with Ninjin’yoeito (NYT), a traditional Kampo medicine, ameliorates anorexia and fatigue [10] and muscle atrophy [11] as well as retaining memory function in old subjects with dementia, and that it improves anorexia [12], [13] and muscle atrophy [14] in mouse models, indicating NYT’s potential against frailty and sarcopenia [15] a.

The present study explored whether NYT alters circadian feeding behavior and thereby controls frailty. NYT was administered by oral gavage to aged and young mice at various time points, followed by measurement of interval food intake for 24 h. Effects of NYT administration for 3 days were assessed on the major components of psychological and physical domains of frailty [1], [2]; anxiety-like behavior with elevated plus maze [16], memory with Y-maze [17], and grip strength with stretcher [18].

## Materials and methods

### Agents

Ninjin’yoeito (NYT), NYT lacking Chenpi (NYT w/o Chenpi), Chenpi, and hesperidin in dried powder were supplied by Kracie Co. (Tokyo, Japan). NYT, Chenpi or hesperidin was mixed with distilled water (DW) to prepare stock solution before each experiment. Ensure H (66 % carbohydrate, 17 % protein, 17 % fat; 1.5 kcal/mL) was purchased from Abbott Japan (Tokyo, Japan).

### Animals

Male C57BL/6 J mice aged 6–12 weeks were obtained from Clea Inc (Osaka, Japan), and those aged 75–90 from Charles River Laboratories (Yokohama, Japan) or National Center for Geriatrics and Gerontology (Obu. Aichi, Japan). They were fed normal diet CE-2 (Clea, Osaka, Japan), and housed under controlled temperature (23 ± 1°C) and humidity (55 ± 5 %), lights on/off at 8:00/20:00, and free access to food and water. Animal experiments were carried out after receiving approval from the Institutional Animal Experiment Committees at Kobe University (IACUC; 30–10–06-R1) and Gifu University (IACUC; 2021–235).

### Administration and dose of substances and measurement of interval food intake

All mice housed in individual cages were subjected to handling for 1 week before experiments. NYT (1 g/kg body weight), NYT w/o Chenpi (1 g/kg), Chenpi (66.7 mg/kg), hesperidin (1 mg/kg), or DW was administered by oral gavage at 8:00, 12:00 or 20:00. Dose of NYT was based on the human dose, 6.7 g per person, and human-to-mice conversion factor 12.3 [19]: 6.7 g/70 kg x 12.3 = 1.18 g/kg, yielding 1 g/kg. Doses of Chenpi and hesperidin were determined by their fractional contents in NYT [20]. Single NYT gavage was followed by measurements of interval and daily food intake.

For measuring interval food intake, the food remaining in feedbox was weighed at specified time point and the difference between two particular time points was considered the interval food intake during this period.

### Protocol for 3 days treatment, pair-fed and functional tests

In other series of experiments, NYT or DW was once daily administered by oral gavage for 3 days, followed by elevated plus maze, Y-maze and grip strength tests on the next day. During the 3 days period, NYT group was pair-fed to control group in 18:00–20:00 by the following protocol. Under food deprivation in 19:00–20:00 in NYT group, the average food intake in 18:00–19:00 in the pair-fed NYT group was almost identical to the average food intake in 18:00–20:00 in control group. Thus, this protocol achieved NYT group’s pair-fed to control group in 18:00–20:00, and was used in pair-fed studies.

### Measurements of plasma hormone levels

NYT administration at 12:00 was followed by blood collection from the tail vein using heparinized capillary glass tubes. Ghrelin was measured with Active-Ghrelin ELISA Kit MM-401 (LSI Medience Co., Tokyo, Japan), Desacyl ghrelin with Desacyl-Ghrelin ELISA Kit MM-402 (LSI Medience Co., Tokyo, Japan), GLP-1 with GLP-1 Total kit K1503 PD (Meso scale discovery, MD, USA), and GIP with Rat/Mouse GIP (total) ELISA Kit EZRMGIP-55K (EMD Millipore, MA, USA).

### Real-time RT-PCR for neuropeptide mRNA expressions in the hypothalamus

After NYT administration at 12:00, mice were deeply anesthetized with 2 % isoflurane and decapitated at 18:00 or 20:00, and their brains were removed. Brain slices were prepared, and entire ARH and PVH were excised from left and right sides. Total RNA was isolated using Trizol (Invitrogen, Carlsbad, CA, USA) and RNase-free DNase (Molecular Bio Products Inc. California, USA). After treatment with RQ1 DNase (Promega, Madison, WI, USA), first-strand cDNA was synthesized with ReverTra Ace kit (Takara Bio-Inc., Shiga, Japan). Using SYBR Premix Ex Taq II, quantification was performed via the ∆∆CT method with Thermal Cycler Dice (Takara Bio-Inc., Shiga, Japan), while glyceraldehyde 3 phosphate dehydrogenase (GAPDH) was used as a control, as previously reported [21]. The primers were listed in Supplementary Table 1.

### Elevated plus maze test

Anxiety-related behavior was assessed by elevated plus maze test [16], using MK-10 (Shinfactory, Fukuoka, Japan). All mice were placed in the central platform facing all arms, and their behavior was recorded for 10 min. The percentage of the time in open arms (%) was used as an anti-anxiety index.

### Y-maze test

Short-term spatial memory was assessed by Y-maze test [17], using MY-10 (Shinfactory, Fukuoka, Japan) with 3 arms A, B and C, each 40 cm long, 20 cm high and 10 cm wide. Each mouse was initially placed in arm A, permitted free exploration for 10 min [22] and the sequence of arm entries was recorded. The working memory capability was determined by the percentage of entry into three different arms in succession, being expressed as the alternation triplet.

### Grip strength test

Forelimb grip strength was measured using Grip Strength Meter GPM-100 (Melquest, Toyama, Japan) [18]. When the mouse's tail is instantaneously pulled, the mouse tightly grasps the bar and the tension (N) was recorded. The average value from 5 trials in each mouse was used.

### Statistical analysis

Data are presented as mean ± SEM. All groups showed normal variance and equal variances as a result of F test or Bartlett’s test. Statistical analysis was performed by two-tailed unpaired or paired *t*-test between 2 groups. In more than three groups, one-way or two-way ANOVA was followed by Sidak's or Tukey's multiple comparisons test. All analyses were performed using Prism 8 (GraphPad Software, CA). p < 0.05 was considered significant.

## Results

### NYT, independently of administration timing, promotes circadian feeding in aged and young mice

**Fig. 1:**
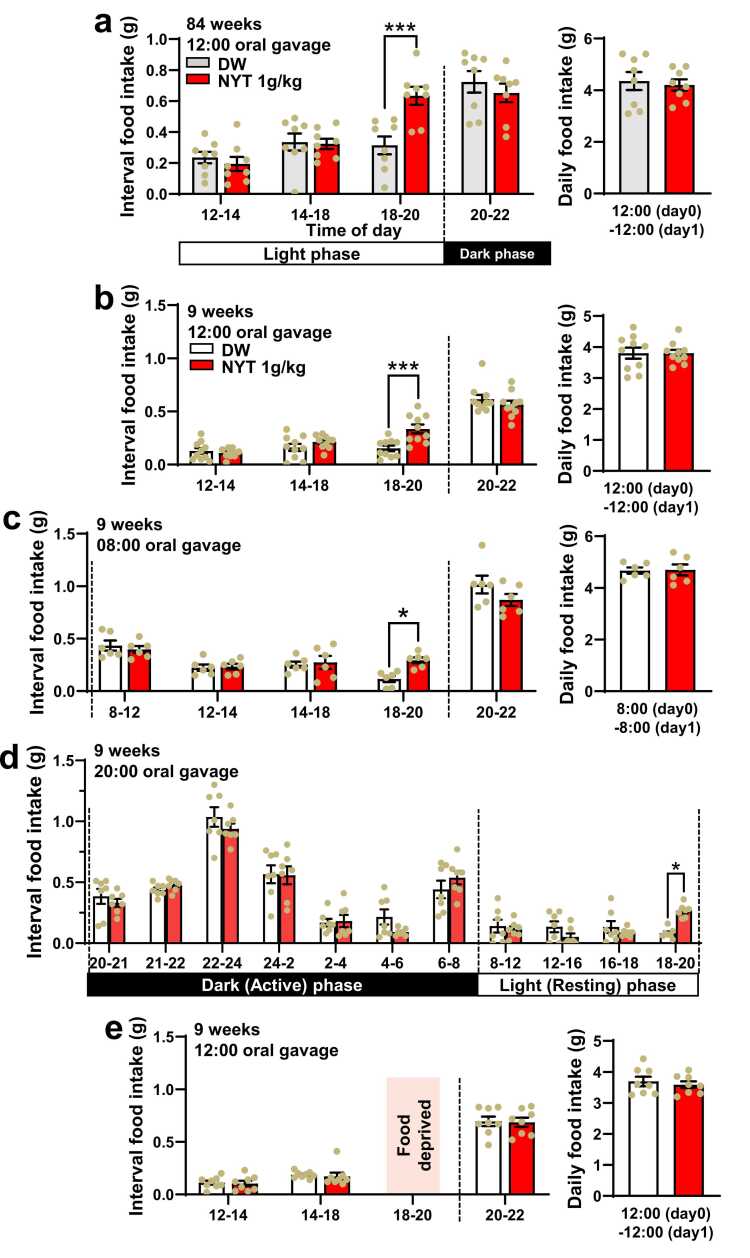
Ninjin’yoeito (NYT), independently of administration timing, promotes pre-active phase feeding in aged and young mice. a-b, In 84-week-old aged mice (a) and 9-week-old young mice (b), NYT administered by oral gavage at 12:00, compared to DW, increased interval food intake in 18:00–20:00 without altering it in other time flames (left panels) and daily food intake (right panels). c-d, NYT administration at 08:00 (c) and 20:00 (d) increased interval food intake specifically in 18:00–20:00 in young mice. e, Under food deprivation selectively in 18:00–20:00 NYT did not alter feeding pattern in young mice. Dots indicate individual data in each mouse. Vertical dotted lines indicate light on/off (08:00/20:00). *p < 0.05, ***p < 0.001 by two-way ANOVA followed by Sidak's multiple comparisons test. n = 8 (a), 10 (b), 6 (c), 7 (d), and 8 (e).

Under light on/off at 08:00/20:00, oral gavage of NYT at 12:00, compared to DW (control), increased interval food intake in 18:00–20:00 without significantly altering it in other time flames and daily food intake in 84-week-old aged mice (Fig. 1a) and 9-week-old young mice (Fig. 1b). The time flame of 18:00–20:00 precedes active phase (20:00–08:00) and is defined as the pre-active phase in this study. NYT administered at 08:00 (Fig. 1c) or 20:00 (Fig. 1d) also increased food intake selectively in 18:00–20:00. Under food deprivation in 18:00–20:00, NYT did not significantly alter food intake in any time period (Fig. 1e). Thus, NYT, regardless of administration timing, increases food intake selectively in pre-active phase in aged and young mice, indicating circadian feeding promotion. The interval food intake in some time periods tended to decrease (Fig. 1a,c,d), which may have cancelled out the increased food intake in 18:00–20:00, resulting in no change in daily food intake.

### NYT elevates anti-anxiety behavior, memory and grip strength via promoting circadian feeding in aged mice

**Fig. 2:**
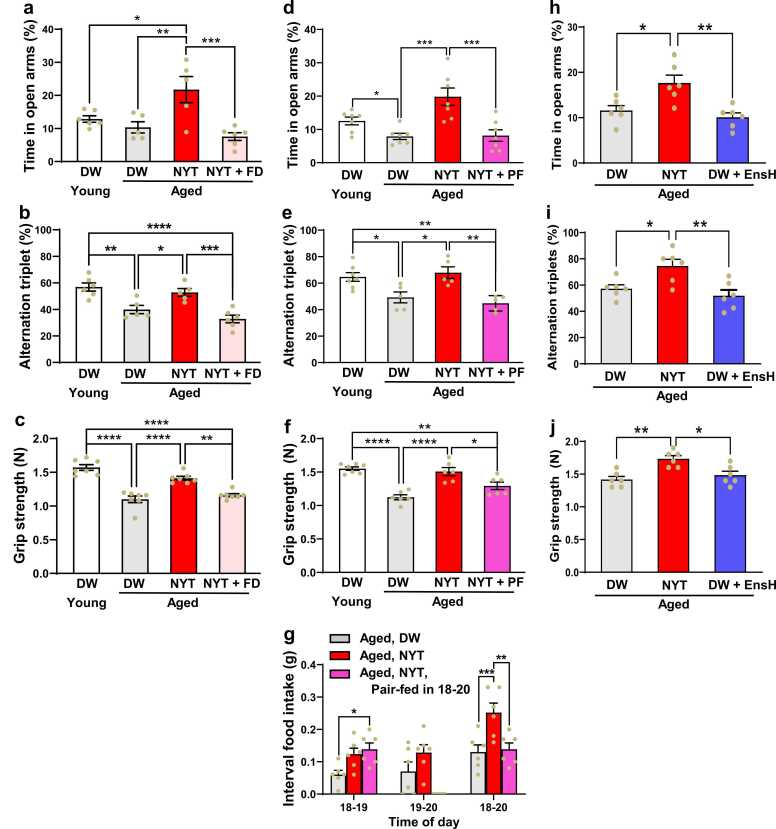
NYT, depending on feeding promotion in 18:00–20:00, elevates physiological functions in aged mice. Oral gavage of NYT and DW at 12:00 for 3 days was followed by assessment of physiological functions on next day. Some NYT-groups were combined with food deprivation (FD) (a-c) or pair-fed (PF) to DW group (d-g) in 18:00–20:00 for 3 days. a-c, In 103-week-old mice, the time in open arms (a), alternation triplet (b), and grip strength (c) declined compared to 9-week-old young mice and markedly increased by NYT. These increases were blocked by food deprivation in 18:00–20:00. d-g, In 84-week-old (d) and 104-week-old (e,f) mice, the time in open arms (d), alternation triplet (e), and grip strength (f) declined compared to young mice and markedly increased by NYT. These increases were blocked by pair-fed. (g) Protocol for pair-fed in 18:00–20:00 by food deprivation in 19–20. n = 5–6. 82-week-old (d), 104-week-old (e,f), and 89-week-old mice (g). h-j, Aged (88–89 weeks) mice were treated with DW, NYT, and DW accompanied by iso-calorie pair-fed to NYT group with adding Ensure H (EnsH) in 18:00–20:00 for 3 days, followed by assessment of physiological functions. *p < 0.05, **p < 0.01, ***p < 0.001, ****p < 0.0001 by one-way ANOVA followed by Tukey's multiple comparisons test. n = 5–6 (a,b), 7 (c,d), 5–7 (e), 6–7 (f), and 6 (g,h,i,j).

In elevated plus maze test, the time in open arms reflecting the anti-anxiety activity [16] tended to be lower in 103-week-old mice compared to 10-week-old mice (Fig. 2a), and markedly elevated after oral gavage of NYT at 12:00 for 3 days (Fig. 2a). This elevation was completely blocked by the food deprivation in 18–20 for 3 days of NYT treatment (Fig. 2a), while this manipulation did not affect diurnal feeding pattern (Fig. 1e). In Y maze test [17], alternation triplet was significantly reduced in aged mice compared to young mice (Fig. 2b), and markedly restored by NYT administration for 3 days. This restoration was completely blocked by food deprivation in 18:00–20:00(Fig. 2b). Grip strength, assessed by grip strength test [18], was significantly reduced in aged mice compared to young mice (Fig. 2c), and improved by NYT treatment. This improvement was markedly attenuated by food deprivation in 18:00–20:00 (Fig. 2c). In 9-week-old young mice, NYT did not affect anxiety-like behavior, memory, and grip strength (Supplementary Fig. 1a-c).

To explore whether the increment of food intake in 18:00–20:00 by NYT is essential for promoting physiological functions, NYT-treated aged mice were pair-fed to control in 18:00–20:00 for 3 days (Fig. 2d-g), as achieved by food deprivation only in 19:00–20:00 (Fig. 2g). The NYT-induced rises in anti-anxiety activity (Fig. 2d) and alternation triplet (Fig. 2e) were completely blocked and rise in grip strength (Fig. 2f) was largely attenuated by the pair-fed. Thus, the NYT-driven feeding promotion at pre-active phase upregulates psychological and physical functions in aged mice, acting against frailty.

The results raised a further question whether the incremental food intake in 18:00–20:00 serves as the sufficient, as well as necessary, condition. The mice receiving DW were administered with Ensue H by oral gavage at 19:50, so that the calorie intake from CE2 and Ensure H was paired to that from CE2 in NYT group in 18:00–20:00. This iso-calorie pair-fed for 3 days showed no changes in anxiety-like behavior, memory, and grip strength (Fig. 2h-j), indicating that the artificially increased energy intake in 18:00–20:00 without NYT is ineffective, suggesting the NYT-triggered appetite-driven rise in intake is essential.

### Effects of NYT on plasma hormones

**Fig. 3:**
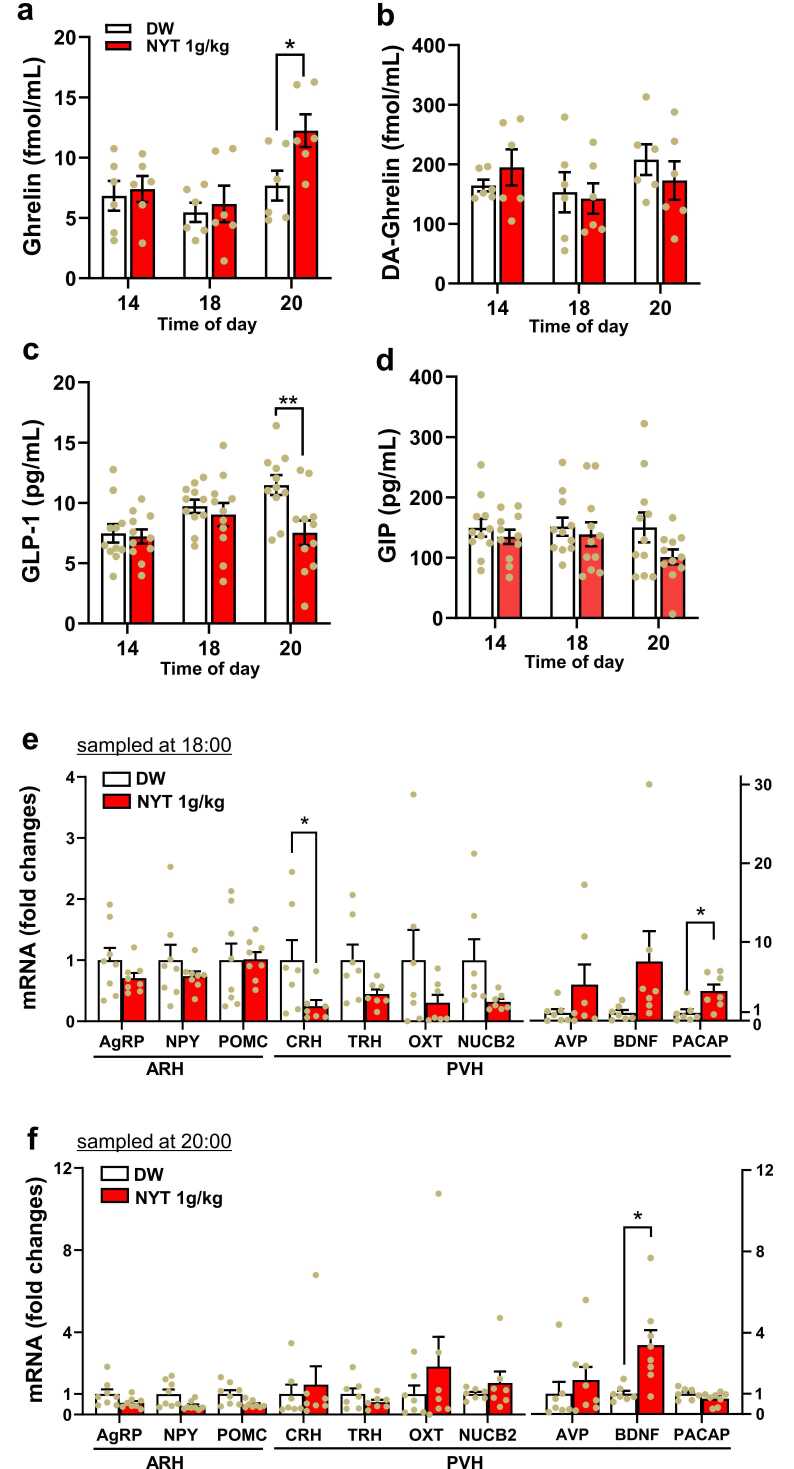
Effects of NYT on plasma hormone levels and hypothalamic neuropeptide mRNA expressions. a-d, Effects of NYT administration at 12:00 on plasma ghrelin (a), desacyl-ghrelin (b), GLP-1 (c), and GIP (d) levels in 9-week-old mice. e-f, Effects of NYT administration at 12:00 on ARH AgRP, NPY, POMC, and PVH CRH, TRH, OXT, NUCB2, AVP, BDNF, and PACAP mRNA expressions (fold change) at 18:00 (e) and 20:00 (f) in 9-week-old mice fasted from 08:00. *p < 0.05 and **p < 0.001 by two-way ANOVA followed by Sidak's multiple comparisons test. n = 6 (a,b), 11 (c,d), and 7–8 (e,f).

NYT administered by oral gavage at 12:00, compared to DW, increased plasma level of orexigenic ghrelin at 20:00 without altering it at 14:00 and 18:00, suggesting increased ghrelin secretion during 18:00–20:00 time frame in 9-week-old mice (Fig. 3a). Plasma desacyl (DA)-ghrelin did not change (Fig. 3b). As for anorexigenic hormones, NYT reduced plasma glucagon-like peptide-1 (GLP-1) level at 20:00 but not 14:00 and 18:00 (Fig. 3c), suggesting decreased GLP-1 secretion during 18:00–20:00, while glucose-dependent insulinotropic polypeptide (GIP) was not altered (Fig. 3d).

### Effects of NYT on hypothalamic neuropeptide mRNA expressions

Effects of NYT on neuropeptide mRNA expressions in the arcuate nucleus (ARH) and paraventricular nucleus of the hypothalamus (PVH) were examined. In the sample at 18:00 (Fig. 3e), oral gavage of NYT at 12:00 significantly decreased corticotropin-releasing hormone (CRH) and elevated pituitary adenylate cyclase-activating peptide (PACAP) mRNA expressions in PVH, without significantly altering those of agouti-related protein (AgRP), neuropeptide Y (NPY) and pro-opiomelanocortin (POMC) in ARH and thyrotropin-releasing hormone (TRH), oxytocin (OXT)**,** nucleobindin-2 (NUCB2), arginine vasopressin (AVP) and brain-derived neurotrophic factor (BDNF) in PVH (Fig. 3e). In the sample at 20:00 (Fig. 3f), CRH and PACAP expressions returned to control levels, while BDNF expression in PVH significantly increased.

### Chenpi and hesperidin mimic NYT in promoting circadian feeding

**Fig. 4:**
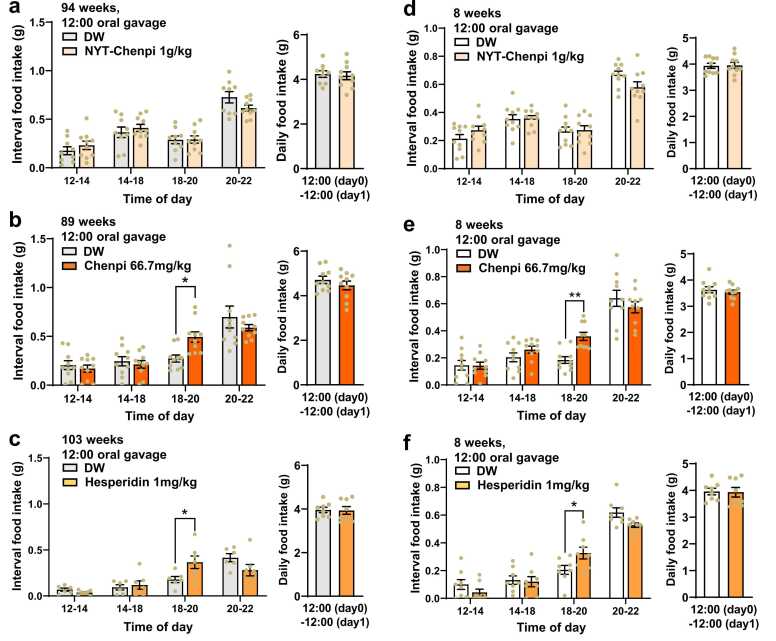
Effect of NYT lacking Chenpi, Chenpi and hesperidin on food intake. NYT lacking Chenpi (NYT w/o Chenpi) failed to alter food intake, but Chenpi and hesperidin increased it selectively in 18:00–20:00 in aged (a-c) and young mice (d-f). Oral gavage of substances at 12:00. 94-week-old (a), 89-week-old (b), 103-week-old (c) and 8-week-old mice (d-f). *p < 0.05, **p < 0.001 by two-way ANOVA followed by Sidak's multiple comparisons test. n = 9–10 (a), 10 (b), 6–7 (c), 10 (d,e), and 8 (f).

Chenpi, one of 12 crude herbs in NYT, and hesperidin, an abundant constituent in Chenpi [23], show orexigenic ability [23], [24]. In 94-week-old aged mice, oral gavage of NYT lacking Chenpi (NYT w/o Chenpi) at 12:00 failed to alter interval food intake in 18:00–20:00 and other time frames (Fig. 4a). By contrast, administration of Chenpi at 12:00 increased food intake specifically in 18:00–20:00 (Fig. 4b). Furthermore, hesperidin, administered by oral gavage at 12:00, increased food intake specifically in 18:00–20:00 (Fig. 4c). Likewise, in 8-week-old young mice, NYT w/o Chenpi failed to alter interval food intake in 18:00–20:00 (Fig. 4d), while Chenpi (Fig. 4e) and hesperidin (Fig. 4f) increased it. These results are consistent with orexigenic properties of Chenpi [24] and hesperidin [24].

## Discussion

This study revealed that NYT, irrespective of administration timing, promotes feeding selectively in pre-active phase and consequently raises anxiety-like behavior, memory and grip strength in aged mice, establishing a concept ‘pre-active phase feeding against frailty’. The key novelty of this study is that it shows when to eat is crucial for psychological and physical activities and the critical timing is pre-active phase. Although a number of human studies have suggested the importance of eating breakfast for health, most of them were cross-sectional and causal association remained unclear [25], [26]. This study presents solid evidence for the key role of pre-active phase feeding promotion in counteracting age-related vulnerability, providing a new avenue for treating frailty by enriching breakfast in older people.

Feeding elevation is thought to maintain physiological activities in older adults with frailty [4], [5], [27], [28]. However, possible role of timing of eating in frailty has remained largely unknown. The present study revealed that feeding promotion in pre-active phase 18:00–20:00 in mice, which corresponds to 06:00–08:00 in humans, counteracts frailty in aged mice. Hence, the translational message to humans is that boosting the early morning appetite to enrich breakfast could intervene frailty.

This study found elevated ghrelin and reduced GLP-1 in plasma as possible drivers for circadian feeding promotions by NYT. The role of ghrelin and GLP-1 has been reported: NYT elevated plasma level of ghrelin and suppressed that of PYY, a peptide secreted with GLP-1 from L-cells, in parallel with amelioration of anorexia in cisplatin-treated mice [13], suggesting that the changes in ghrelin and GLP-1 improve appetite. Furthermore, hesperidin elevates appetite possibly by promoting ghrelin release via antagonizing serotonin receptor and by promoting ghrelin action [29], suggesting an orexigenic route via hesperidin-serotonin-ghrelin system. Whether NYT/hesperidin can directly act on ghrelin-secreting and/or GLP-1-secreting cells remains to be studied.

NYT irrespective to administration timing increased food intake specifically in 18:00–20:00. The increased food intake at the end of the resting period when feeding activity is low fits with previous report that NYT modulates feeding under negative energy balance [30]. Although the underlying mechanism remains to be clarified by future studies, we propose the following two-step model. Exogenous NYT gavage makes a yet-unidentified alteration in the body, including the brain, peripheral tissues, and/or gut microbiota. Microbiota is regulated by some of NYT’s constituents [31] and plays a role in the clock-nutrition interplay [8]. This alteration does not immediately influence feeding but persist, and interacts with circadian clock-driven endogenous changes occurring in pre-active phase, leading to circadian feeding promotion via mechanisms including changes in neurohormones in pre-active phase: elevated ghrelin that initiates food ingestion [32], [33], elevated PVH PACAP that drives orexigenic neuronal transmission to ARH [34], [35], reduced GLP-1 that induces post-prandial satiety [33], and reduced PVH CRH that mediates anxiety/stress-induced anorexia [36]. Notably, PVH is an area implicated in both circadian and feeding regulation [21], [34]. These effects of NYT could involve its constituent hesperidin, which passes through BBB to influence the brain [37].

The sympathetic nerve could play a role in the NYT- and/or hesperidin-induced circadian feeding promotion. Sympathetic nervous activity is controlled by circadian rhythm, rising in the early active phase to increase blood pressure, heart rate, blood glucose, body temperature and appetite in early morning in humans [38]. This sarge is driven by the circadian sympathetic cascade involving the SCN-PVH circuit [39], [40], [41]. Notably, sympathetic nerve is a potent stimulator of ghrelin secretion form the stomach X/A-like cells [42] and inhibitor of GLP-1 secretion from the intestinal L-cells [43]. Our results, together with these reports, suggest a key role of the circadian sympathetic cascade in the elevated ghrelin, reduced GLP-1 and promoted feeding in pre-active phase. Notably, the present study identified PVH as a principle target of NYT. Accordingly, NYT and/or hesperidin may interact with PVH to advance the rise of the circadian sympathetic cascade, thereby advancing the onset of ghrelin elevation, GLP-1 reduction and appetite promotion in pre-active phase. In addition, it has been reported that NYT excites dopamine neurons in the ventral tegmental area [44] and that dopamine system regulates circadian food anticipatory activity in rats [45], suggesting an implication of dopamine in linking NYT to circadian feeding promotion. How the feeding promotion at pre-active phase is linked to psychological/physical functions remains to be studied. We can speculate that the feeding promotion at pre-active phase nourishes the muscle and brain to enhance their activities during the subsequent active phase. Secondly, elevated feeding behavior at pre-active phase might stimulate the oral-gut tract to emit hormonal, neural, immune, and/or microbiome signals that vitalize remote organs during active phase. Furthermore, in considering that food, as well as light, is a potent entrainer of circadian clock systems [8], feeding promotion in pre-active phase could synchronize the body’s circadian rhythm that is often impaired in aging [9].

The results with food deprivation and pair-fed and with intra-stomach nutrient injection indicated that the incremental food intake in 18:00–20:00 was necessary but not sufficient for elevating psychological/physical functions, suggesting additional permissive factor(s) that cooperate with incremental intake to elevate psychological/physical functions in aged mice. First, the injection of the additional food by mouth may collaborate with incremental intake to elevate physiological functions. In consistent with this, oral functions such as chewing and swallowing can improve impaired physiological functions in aging [46], [47]. Second, the insulin resistance, which often underlies the impaired physiological functions in aging, is reportedly improved by NYT [48]. Third, NYT-induced changes in stress/anxiety/memory-related neurohormones may serve as permissive factors, which include ghrelin [36], [49], CRH-glucocorticoid [36], [50], PACAP [34], and BDNF [51]. The neural effects of NYT might implicate hesperidin that passes through BBB [37] and exerts antianxiety-depressant actions [52].

## Conclusion

This study demonstrated that the NYT-triggered appetite-driven feeding promotion in pre-active phase upregulates psychological and physical abilities that decline in aged mice, establishing a concept ‘pre-active phase feeding against frailty’. Translationally, promoting the early morning appetite and breakfast could provide a novel and efficacious avenue for intervening frailty in older people. It can be achieved with NYT taken as medicine at any time of day and possibly with Chenpi and/or hesperidin, rich substances in citrus [24], as foods and/or supplements.

## Limitation

Experiments were performed with male mice only. Considering reported sex differences in feeding, aging and neurohormonal systems, possible sex differences in effects and involvement of sex hormones remain to be studied.

It is well recognized that estrogen inhibits food intake, whereas testosterone may stimulate appetite [53]. The anorexigenic ability of estrogen is related to gut hormones [54]: estrogen attenuates ghrelin's effect on feeding in ovariectomized animals [53] and the reward feeding in ovariectomized rats is effectively reduced by GLP-1 agonist [55]. Furthermore, PVH is a target for anorexigenic effect of estrogen [53], [56]. Notably, we report that NYT promotes circadian feeding via mechanisms involving ghrelin, GLP-1 and PVH, on which estrogen appears to act. Hence, the NYT-induced circadian feeding may have distinct, as well as common, properties between female and male, and moreover differ in women depending on menstrual cycles and after menopause. This important issue remains to be addressed by future studies.

## Funding

This work was supported by Grant-in-Aid for Scientific Research (C) (25K10163) from Japan Society for the Promotion of Science (10.13039/501100001691JSPS), COMIT Collaborative Research 2024, and Japan Association for Diabetes Education and Care (JADEC) 2024 to T.Y.

## CRediT authorship contribution statement

**Wanxin Han:** Methodology, Investigation. **Boyang Zhang:** Methodology, Investigation. **Hitoshi Kuwata:** Methodology, Investigation. **Yutaka Seino:** Supervision. **Katsuya Dezaki:** Methodology, Investigation. **Daisuke Yabe:** Supervision. **Masanori Nakata:** Methodology, Investigation. **Shin Tsunekawa:** Supervision. **Yermek Rakhat:** Methodology, Investigation. **Yusaku Iwasaki:** Methodology, Investigation. **Lei Wang:** Writing – original draft, Methodology, Investigation. **Takashi Sakurai:** Methodology, Investigation. **Toshihiko Yada:** Writing – original draft, Investigation, Funding acquisition, Conceptualization. **Noboru Ogiso:** Methodology, Investigation. **Chikara Abe:** Methodology, Investigation. **Seiya Banno:** Methodology, Investigation.

## Declaration of Competing Interest

The authors declare the following financial interests/personal relationships which may be considered as potential competing interests: Toshihiko Yada reports financial support and equipment, drugs, or supplies were provided by Kracie Ltd. If there are other authors, they declare that they have no known competing financial interests or personal relationships that could have appeared to influence the work reported in this paper.

## Data Availability

Data will be made available on reasonable request.

## Appendix A Supplementary material

Supplementary material. **Lack of effect of NYT on physiological functions in young mice.** NYT administration at 12:00 for 3 days did not affect anti-anxiety behavior (a), memory (b) and grip strength (c) in 9-week-old young mice. n = 5–7. n = 6 (a,b) and 7 (c)

## Footnotes

Appendix A Supplementary data associated with this article can be found in the online version at doi:10.1016/j.jphyss.2026.100062.
