# Supplementary figures and images for: Circadian feeding promotion by Ninjin’yoeito counteracts frailty in aged mice
Source: J Physiol Sci. 2026 Jan 27;76(1):100062. doi: 10.1016/j.jphyss.2026.100062 (PMC12874285; doi:10.1016/j.jphyss.2026.100062)

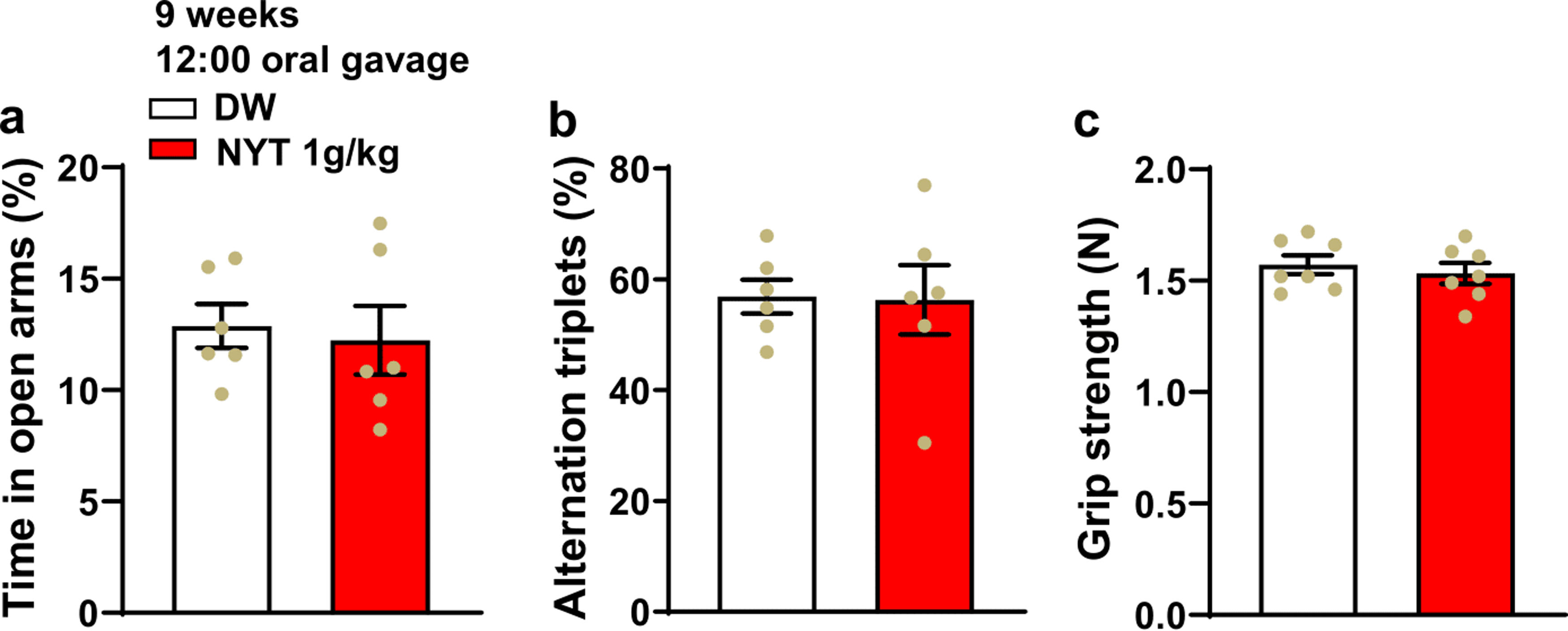

Supplement: Supplementary file 1 — Supplementary material. Lack of effect of NYT on physiological functions in young mice. NYT administration at 12:00 for 3 days did not affect anti-anxiety behavior (a), memory (b) and grip strength (c) in 9-week-old young mice. n = 5–7. n = 6 (a,b) and 7 (c) [file mmc1.jpg]

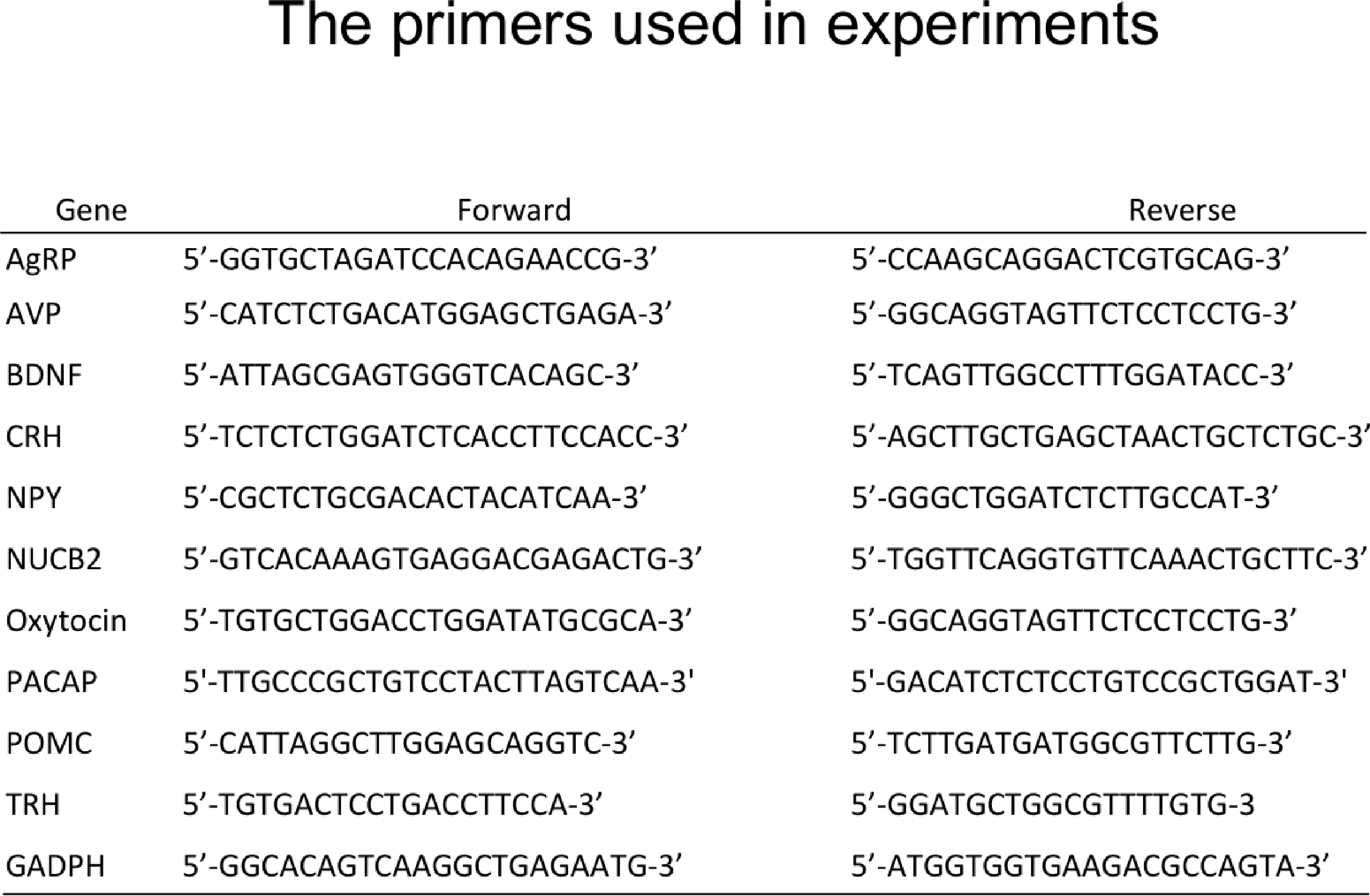

Supplement: Supplementary file 2 — Supplementary material [file mmc2.jpg]
